# Assessing the Influence of HGT on the Evolution of Stress Responses in Microbial Communities from Shark Bay, Western Australia

**DOI:** 10.3390/genes14122168

**Published:** 2023-12-01

**Authors:** Emilie J. Skoog, Gregory P. Fournier, Tanja Bosak

**Affiliations:** 1 Department of Earth, Atmospheric and Planetary Sciences, Massachusetts Institute of Technology, Cambridge, MA 02139, USA; g4nier@mit.edu (G.P.F.); tbosak@mit.edu (T.B.); 2 Scripps Institution of Oceanography, University of California San Diego, La Jolla, CA 92037, USA

**Keywords:** microbial adaptation, molecular evolution, horizontal gene transfer, metagenome-assembled genomes, microbial mat ecology, microbial phylogeny

## Abstract

Pustular microbial mats in Shark Bay, Western Australia, are modern analogs of microbial systems that colonized peritidal environments before the evolution of complex life. To understand how these microbial communities evolved to grow and metabolize in the presence of various environmental stresses, the horizontal gene transfer (HGT) detection tool, MetaCHIP, was used to identify the horizontal transfer of genes related to stress response in 83 metagenome-assembled genomes from a Shark Bay pustular mat. Subsequently, maximum-likelihood phylogenies were constructed using these genes and their most closely related homologs from other environments in order to determine the likelihood of these HGT events occurring within the pustular mat. Phylogenies of several stress-related genes—including those involved in response to osmotic stress, oxidative stress and arsenic toxicity—indicate a potentially long history of HGT events and are consistent with these transfers occurring outside of modern pustular mats. The phylogeny of a particular osmoprotectant transport gene reveals relatively recent adaptations and suggests interactions between Planctomycetota and Myxococcota within these pustular mats. Overall, HGT phylogenies support a potentially broad distribution in the relative timing of the HGT events of stress-related genes and demonstrate ongoing microbial adaptations and evolution in these pustular mat communities.

## 1. Introduction

Pustular microbial mats are complex benthic microbial communities bound to sediments by an organic matrix. Microbial communities residing in pustular mats from Shark Bay, Western Australia experience harsh environmental conditions including hypersalinity, UV radiation, desiccation, and heavy metal toxicity [1,2,3,4,5,6,7,8]. Similar microbial mats have colonized peritidal environments for over two billion years [9,10]. The successful survival of these communities depends on the molecular adaptations and evolutionary responses of the organisms that occupy these mats to these harsh environmental conditions. To combat osmotic stresses, organisms in mats accumulate inorganic ions (“salt-in-cytoplasm” mechanism) and other compatible solutes such as glycine betaine, trehalose, and proline [11,12,13,14,15]. Various community members also detoxify heavy metals and combat oxidative stress to resist cellular damage caused by reactive oxygen species, free radicals, peroxides, and heavy metals [7,16,17].

Genetic plasticity is key in shaping an effective response of microbial communities to these environmental stressors. Horizontal gene transfer (HGT) between organisms is a mechanism by which microbes can maintain genetic plasticity and adapt to environmental conditions. HGT can be mediated by transformation (i.e., uptake of eDNA), conjugation (i.e., transfer of genes through cell-to-cell plasmid exchange), or transduction (i.e., infection by phage). The role of HGT in the evolution of antimicrobial resistance has been particularly well studied, and biofilms have been identified as hot spots for the horizontal transfer of antibiotic resistance genes [18,19,20,21], especially in comparison to planktonic communities [22,23]. Previous studies have also identified the importance of the horizontal transfer of metal resistance genes in the adaptation and evolution of microbial communities from arsenic-rich acid mine drainages, gold mines, and groundwater environments [24,25,26]. HGT events have also been identified to play a role in the adaptation and speciation of archaea, enabling the acquisition of genes related to oxidative stress resistance (e.g., catalase, DNA-binding protein from starved cells) from acidophilic bacteria [27]. However, the contributions of HGT to the adaptation and evolution of microbial communities in microbial mats remain to be assessed. 

High-throughput shotgun metagenomic sequencing has enabled the profiling of natural communities across environments and can provide insight into microbial community interactions, metabolism, and responses to environmental conditions. The identification of HGT events between metagenome-assembled genomes (MAGs) within a metagenomic community can reveal important information regarding community-level adaptations and interactions. Several bioinformatic approaches have been developed for identifying HGT events within metagenomic sequencing data [28,29,30,31,32]. MetaCHIP [31] has been utilized as a stand-alone tool for identifying HGT events, specifically between MAGs in a natural community, and has been used to identify HGT events within various metagenomic communities [33,34,35,36,37,38,39]. However, the methods by which HGT events are determined within metagenomic datasets are continuously developing and there are currently no clear best practices [40].

In this work, we investigate the horizontal transfer of genes associated with stress resistance and response to osmotic stress, oxidative stress, and arsenic toxicity within a microbial metagenome from Shark Bay, Western Australia. We use MetaCHIP to identify HGT events between the 83 MAGs assembled from this microbial mat and determine the efficacy of this tool by coupling maximum-likelihood phylogenetic approaches to these analyses. We use these phylogenetic inferences to confirm the transfer of these genes between the organisms within the microbial mat community, determine the relative timing of numerous transfer events between these MAGs, and explore the evolutionary history of microbial interactions based on the placement of horizontally transferred genes within these phylogenetic trees.

## 2. Materials and Methods

### 2.1. Sample Procurement

Pustular mats from Carbla Beach, Shark Bay, Western Australia, were sampled and sequenced according to Skoog et al. (2022). Briefly, whole genomic DNA was extracted from a pustular mat and metagenomes were sequenced using an Illumina NextSeq sequencer (Cambridge, MA, USA). Resulting DNA sequences were quality filtered, assembled, binned, and taxonomically classified as previously described [5]. This analysis yielded 83 MAGs, which this work utilizes for all subsequent analyses. From here on, they are referred to as ‘MAG X’, where ‘X’ is 1–83. All MAGs are publicly available on Zenodo (https://doi.org/10.5281/zenodo.3874996, accessed on 28 November 2023) and on the Joint Genome Institute website under the GOLD AP ID Ga0316160.

### 2.2. HGT Predictions

**Figure 1:**
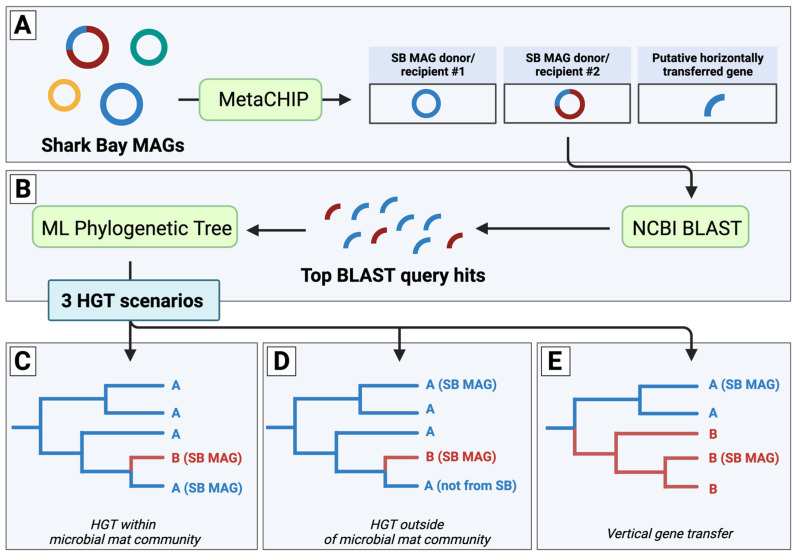
Schematic of HGT detection workflow and possible gene transfer scenarios. (**A**) Putative horizontally transferred genes among Shark Bay (SB) metagenome-assembled genomes (MAGs) are identified by MetaCHIP and (**B**) used as queries within NCBI BLAST to identify top hits for maximum-likelihood (ML) phylogenetic analysis. ML phylogenetic trees may identify three scenarios: (**C**) HGT within the SB microbial mat community, (**D**) HGT outside of the SB microbial mat community, or (**E**) vertical gene transfer.

**Figure 2:**
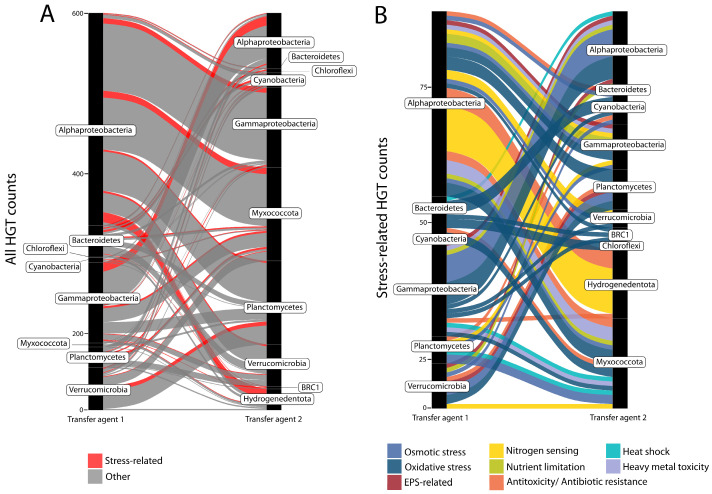
HGT transfers between MAGs of different phyla in a pustular mat from Shark Bay. Alluvial plots show: (**A**) All HGT counts. Genes related to stress are shown in red. (**B**) Predicted HGT events of stress-associated genes only. Different colors indicate the type of stress.

MetaCHIP (v1.10.10) was used to predict HGT events among all MAGs within the pustular microbial mat community at the taxonomic class level ([31]; Figure 1A and Table S1). MetaCHIP uses best-hit analysis between defined phylogenetic/taxonomic groups to identify candidate genes for HGT. The candidate events are subsequently refined and tested using phylogenetic analysis and the reconciliation of species and gene trees. All MAGs were at least 10% above the 40% MAG completeness requirement of the program. Results were plotted using igraph (https://igraph.org/, accessed on 28 November 2023) and ggplot2 in R v.3.6.0 (Figure 2 and Figure S1). All identified genes were then annotated with Prokka (v1.14.6; [41]). 

### 2.3. Phylogenomic and HGT Analyses

All stress-related proteins identified by MetaCHIP as involved in HGT events were selected and used as query sequences to identify the top 100 hits using the NCBI Basic Local Alignment Search Tool (BLAST) with default search parameters (Figure 1B). These proteins were also used as query sequences to identify hits in the unbinned pustular mat metagenome. All sequences maintained a query cover of at least 80%, a minimum e-value of e^−45^, and a percent identity of at least 46%. Seqkit was used to remove any duplicate sequences [42]. Remaining sequences were aligned with MUSCLE (v3.8.31; Ref. [43]). Maximum-likelihood phylogenetic trees were generated using IQTree (v1.6.3) run with ModelFinder Plus (MFP) testing the following base models: LG+, WAG+ and BLOSUM62 (Figure 1B; Ref. [44]). Support for bipartitions was determined using rapid bootstraps (1000 replicates) and SH-aLRT tests (1000 replicates). Trees were mid-point rooted, and FigTree (v.1.4.3) was used to visualize the resulting trees and identify branch lengths for genes that were horizontally transferred. Averaged branch lengths between identified transfer agents (Table S2) were used to construct a histogram and bin widths were established using the Freedman–Diaconis rule [45].

## 3. Results

When making assertions about HGT events within a specific microbial community dataset, it is crucial to differentiate whether these events actually transpired within the analyzed microbial community or are remnants of more ancient transfers. Failing to do so can result in misleading interpretations of the timing of adaptations and evolutionary processes occurring within a given studied community, and it also has the potential to erroneously imply interactions (assuming HGT occurs between organisms in close proximity) among the constituent organisms. In order to assess HGT events and determine whether or not they occurred within the pustular mat community in this study, we first utilized MetaCHIP to identify potential HGTs, and then used these results to construct finer-resolution, maximum-likelihood phylogenies incorporating data outside of the microbial mat community. MetaCHIP predicted 631 gene transfers between 14 different taxonomic classes in the analyzed metagenomic dataset from the pustular mat (Figure S1). Of these predicted transfers, 89 (14%) were of genes that represented 31 different proteins associated with stress responses (Table S1 and Figure 2).

Because MetaCHIP only identifies genes as putatively transferred within a metagenomic community, we constructed the maximum-likelihood phylogenies for each of the 31 different stress-related genes using publicly available gene sequences outside of our Shark Bay community. Incorporating sequences outside of a given dataset provides a more complete evolutionary history and more accurately represents potential HGT events. In analyzing the transfer of a gene between an organism from ‘class A’ and an organism from ‘class B’, we expected the data to yield one of three scenarios. In Scenario 1, the genes identified by MetaCHIP as horizontally transferred between an organism from ‘class A’ and an organism from ‘class B’ would be sister to one another in the maximum-likelihood phylogenetic tree (Figure 1C). This would strongly indicate that the gene transfer occurred between these two organisms within the pustular mat community. In Scenario 2, the gene from ‘class A’ would be sister to a gene from ‘class B’ from an organism not identified within the pustular mat community (Figure 1D). This would suggest that there was an HGT event that occurred; however, this transfer likely did not occur within the microbial mat. Instead, this transfer may have taken place prior to the establishment of the ‘class B’ organism within the pustular mat. In Scenario 3, the gene from ‘class A’ and ‘class B’ are identified within a group of other genes from organisms of ‘class A’ and ‘class B’, respectively (Figure 1E). This would indicate that these genes identified by MetaCHIP as horizontally transferred within the microbial mat were, in fact, vertically transferred.

Of the 31 constructed maximum-likelihood phylogenies, 18 trees fell into Scenario 3 and supported vertical gene transfer phylogenies (Figure 1E). The remaining 13 trees revealed HGT events which fell into either Scenario 1 or Scenario 2 (Figure 1C,D). These trees identified transfer events involving genes related to osmotic stress (i.e., glycine/sarcosine N-methyltransferase, osmoregulated proline transporter, trehalose transport system permease protein, trehalase), oxidative stress (i.e., catalase-peroxidase, chaperone protein, rubrerythrin), arsenic toxicity (i.e., arsenate reductase, arsenite efflux permease), nutrient limitation (i.e., DNA protection during starvation protein, polyphosphate phosphotransferase), and antibiotic resistance (i.e., multidrug export ATP-binding/permease, N-ethylmaleimide reductase). Of these, we explored gene transfer events related to osmotic stress, oxidative stress, and arsenic toxicity in more detail as these are especially relevant stresses faced by microbial community members in Shark Bay.

### 3.1. Osmotic Stress

**Figure 3:**
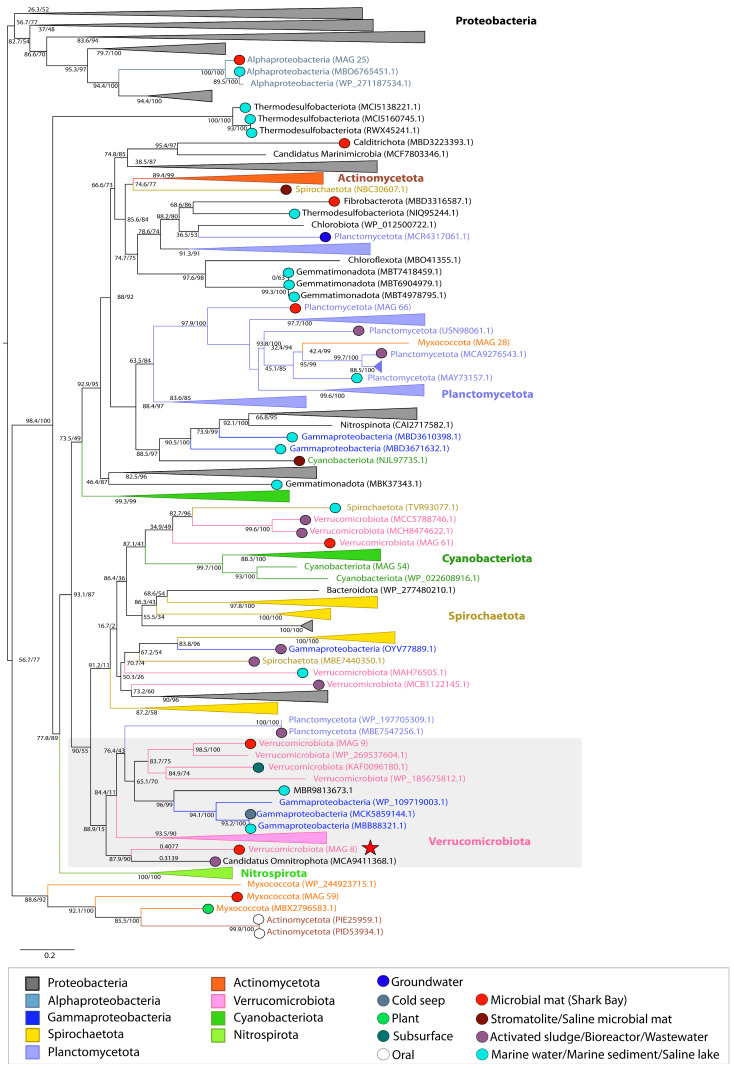
Midpoint-rooted maximum likelihood tree of glycine/sarcosine N-methyltransferase (GSMT) homologs. Support values indicate approximate likelihood ratio test (aLRT)/ bootstrap (100 replicates). The full tree contains 317 unique sequences. Collapsed groups are labeled with taxonomic group names and are colored according to the legend. Circles on branch tips are present and colored according to the sample origin, when data was available. MAGs from the Shark Bay microbial mat metagenome are identified in parentheses as ‘MAG X’. Predicted HGT events involving these MAGs are delineated by a red star. Branch lengths for taxa involved in HGT events are listed above the leaves.

Organisms adapt to osmotic stress in hypersaline environments by producing and importing compatible solutes such as glycine betaine, proline, and trehalose [46]. Glycine/sarcosine N-methyltransferase (GSMT) plays an important role in the production of the osmoprotectant glycine betaine [47]. MetaCHIP analysis revealed the horizontal transfer of this gene among Verrucomicrobia, Planctomycetes, Alphaproteobacteria, Myxococcota, and Cyanobacteria (Table S1). GSMT phylogeny identified Proteobacteria as the most deeply branching group and the remaining tree topology reflected a polyphyletic distribution of these proteins, consistent with evolutionary histories of HGT (Figure 3). The GSMT from Alphaproteobacteria MAG 25 was nested within the most deeply branching group of GSMT genes from Proteobacteria and its closest homologous sequence was identified in an Alphaproteobacterium from the marine environment (Figure 3). GSMT sequences from all other MAGs placed within the polyphyletic portion of the tree. The genes from Planctomycetota MAG 66 and Cyanobacteria MAG 54 were placed within clusters of Planctomycetota and Cyanobacteria sequences, respectively, whereas the genes from Verrucomicrobia MAG 9 and MAG 61 both were identified in separate groups of Verrucomicrobia in this polyphyletic region (Figure 3). GSMT from Myxococcota MAG 28 was the only Myxococcota GSMT present within a cluster of Planctomycetes, suggesting its potential acquisition from this group. In contrast, the GSMT from Myxococcota MAG 59 resided in a more deeply branching group comprised of other Myxococcota and Actinomycetota. The GSMT from Verrucomicrobia MAG 8 was identified on a well-supported (87.9/90) branch sister to a sequence from Candidatus Omnitrophota (MCA9411368.1), indicating a likely gene transfer event between these two organisms (Figure 3). The presence of other Verrucomicrobia outgroups in this cluster (shaded) and the absence of other sequences from Candidatus Omnitrophota support the likely transfer of this protein from a Verrucomicrobia to a Candidatus Omnitrophota organism (Figure 1D and Figure 3).

**Figure 4:**
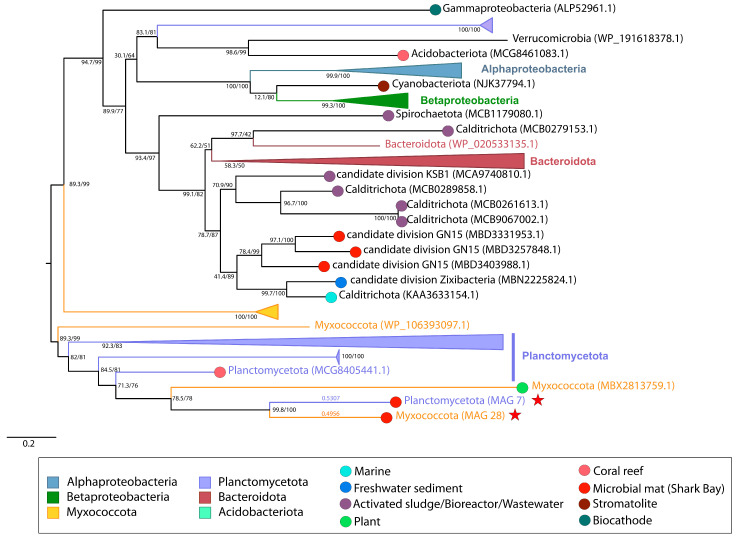
Midpoint-rooted maximum likelihood tree of osmoregulated proline transporter (*opuE*) homologs. Support values indicate approximate likelihood ratio test (aLRT)/bootstrap (100 replicates). The full tree contains 122 unique sequences. Collapsed groups are labeled with taxonomic group names and are colored according to the legend. Circles on branch tips are present and colored according to the sample origin, when data was available. MAGs from the Shark Bay microbial mat metagenome are identified in parentheses as ‘MAG X’. Predicted HGT events involving these MAGs are delineated by a red star. Branch lengths for taxa involved in HGT events are listed above the leaves.

The high-affinity proline transporter, *opuE*, is inducible under hyperosmotic conditions and allows the import of exogenous proline, an osmoprotectant [48,49,50,51]. MetaCHIP analyses identified the transfer of *opuE* between Planctomycetota MAG 7 and Myxococcota MAG 28 from the pustular mat. Maximum-likelihood phylogeny of *opuE* revealed MAG 7 and MAG 28 as well supported (99.8/100) sisters to one another, supporting the hypothesis that *opuE* was transferred between these two MAGs *within* the microbial mat community (Figure 1C and Figure 4). Because more distal groups include both Planctomycetota and Myxococcota, the direction of transfer could not be resolved.

**Figure 5:**
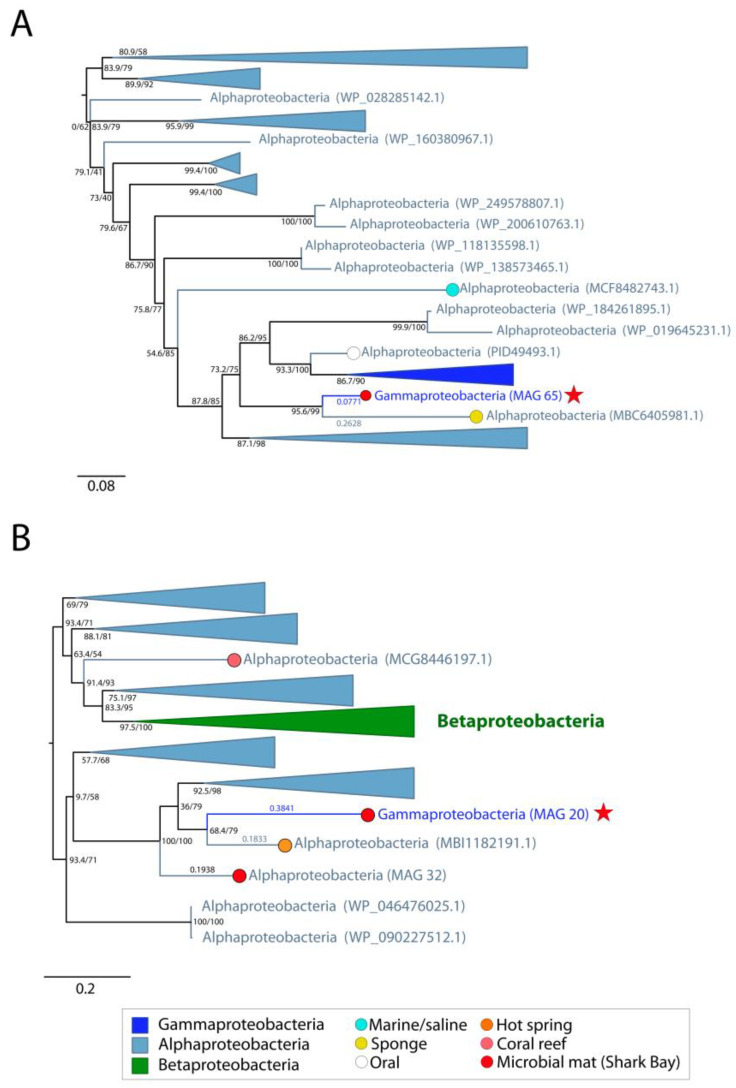
Midpoint-rooted maximum likelihood tree of (**A**) trehalase and (**B**) trehalose transport system permease protein homologs. Support values indicate approximate likelihood ratio test (aLRT)/bootstrap (100 replicates). The full trehalase tree contains 111 unique sequences and the full trehalose transport protein tree contains 195 unique sequences. Collapsed groups are labeled with taxonomic group names and are colored according to the legend. Circles on branch tips are present and colored according to the sample origin, when data was available. MAGs from the Shark Bay microbial mat metagenome are identified in parentheses as ‘MAG X’. Predicted HGT events involving these MAGs are delineated by a red star. Branch lengths for taxa involved in HGT events are listed above the leaves.

Trehalose transporters similarly assist in intracellular resistance to osmotic stress under varying salt concentrations. MetaCHIP results suggested the transfer of a trehalose transporter protein between Alphaproteobacteria MAG 16 and Gammaproteobacteria MAG 65, and identified the transfer of a trehalase (E.C.3.2.1.28) protein, involved in the metabolism of trehalose [52], between Alphaproteobacteria MAG 16 and Gammaproteobacteria MAG 20. Maximum-likelihood phylogenies of both the trehalose transporter and trehalase proteins revealed Alphaproteobacteria as the most deeply branching group. Clusters of Gammaproteobacteria and Betaproteobacteria were also present within the trehalose transporter and trehalase trees, respectively (Figure 5). Alphaproteobacteria MAG 16 was monophyletic with Alphaproteobacteria groups in both trees. The phylogeny of the trehalose transporter protein identified Gammaproteobacteria MAG 65 as sister to an Alphaproteobacterium (MBC6405981.1) from a marine sponge (Figure 5A), whereas the phylogeny of trehalase revealed the sequence from Gammaproteobacteria MAG 20 as sister to the sequence from an Alphaproteobacterium (MBI1182191.1) identified in a hot spring environment (Figure 5B). This supports the horizontal transfer of these genes between MAGs from the pustular mats and organisms outside of these mats (Figure 1D). 

### 3.2. Oxidative Stress

**Figure 6:**
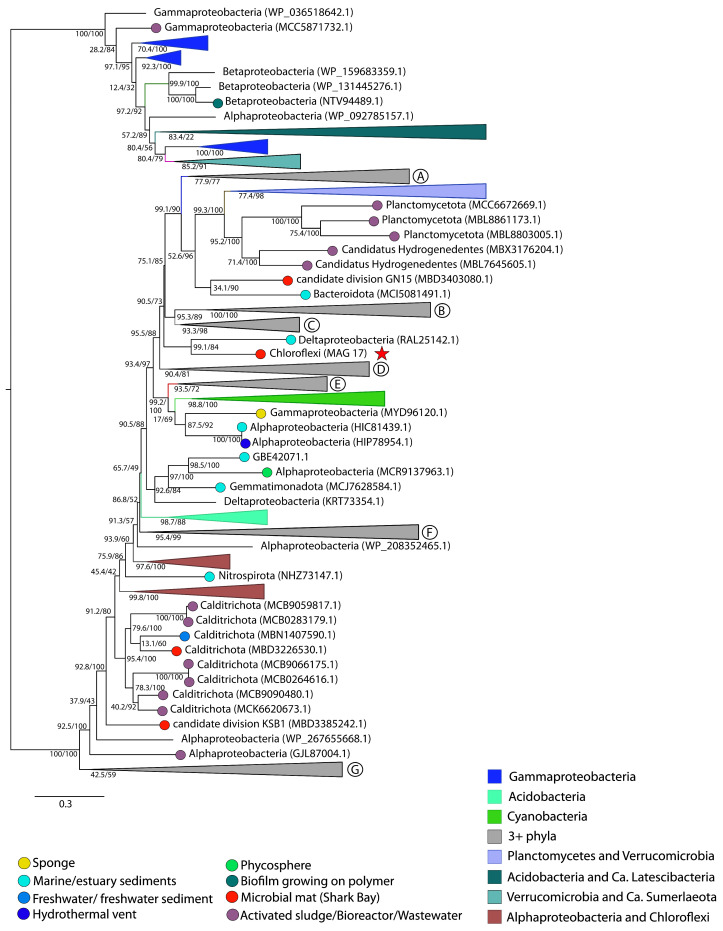
Midpoint-rooted maximum likelihood tree of catalase-peroxidase homologs. Support values indicate approximate likelihood ratio test (aLRT)/bootstrap (100 replicates). The full tree contains 1504 unique sequences. Collapsed groups are labeled with taxonomic group names and are colored according to the legend. Collapsed groups that contain more than three different phyla were noted with circled letters and constitute the following groups: (A) Myxococcota, Acidobacteria, Deltaproteobacteria, Candidatus Latescibacteria (B) Bacteroidetes, Verrucomicrobia, Gammaproteobacteria, Deltaproteobacteria, Spirochaetes, Thermodesulfobacteria, Ca. Dadabacteria, Ignavibacteria, Balneolaeota (C) Alphaproteobacteria, Gammaproteobacteria, Betaproteobacteria, Chloroflexi, Acidobacteria, Euryarchaetoa, Firmicutes, Bacteroidetes (D) Alphaproteobacteria, Chloroflexi, Gemmatimonadetes, Cyanobacteria (E) Alphaproteobacteria, Gammaproteobacteria, Bacteroidetes, Candidatus Omnitrophica (F) Alphaproteobacteria, Betaproteobacteria, Deltaproteobacteria, Gammaproteobacteria, Chloroflexi, Bacteroidetes, Ignavibacteria, Acidobacteria, Planctomycetes, two unbinned sequences (G) Alphaproteobacteria, Betaproteobacteria, Gammaproteobacteria. Legend shows the coloring of circles on branch tips according to the sample origin, when data was available. MAGs from the Shark Bay microbial mat metagenome are identified in parentheses as ‘MAG X’. Predicted HGT events involving these MAGs are delineated by a red star.

Pustular microbial mat communities in peritidal environments produce reactive oxygen species (ROS) and are consistently exposed to these molecules. Electron transport chains involved in microbial photosynthesis and aerobic metabolisms as well as exogenous sources such as xenobiotics and UV radiation can all generate ROS [7,53,54]. An imbalance between the production and removal of ROS can cause irreversible oxidative damage of cellular material [55,56,57], so microorganisms employ various mechanisms to combat oxidative stress, including using enzymes such as catalases, peroxidases, catalase-peroxidases, and rubrerythrin (RBR) [54]. MetaCHIP results suggested the transfer of 13 catalase-peroxidase genes among 9 different phyla within the pustular mat community (Table S1). Maximum-likelihood phylogeny revealed that 12 of these catalase-peroxidases were vertically inherited (Figure 1E) and the catalase-peroxidase of Chloroflexi MAG 17 constituted the only HGT event (Figure 1D). This sequence was placed sister to a sequence from a marine Deltaproteobacterium (Figure 6). All other sequences predicted as HGT by MetaCHIP were instead nested within the sequences from the same phyla, suggesting vertical inheritance (Figure 1E).

**Figure 7:**
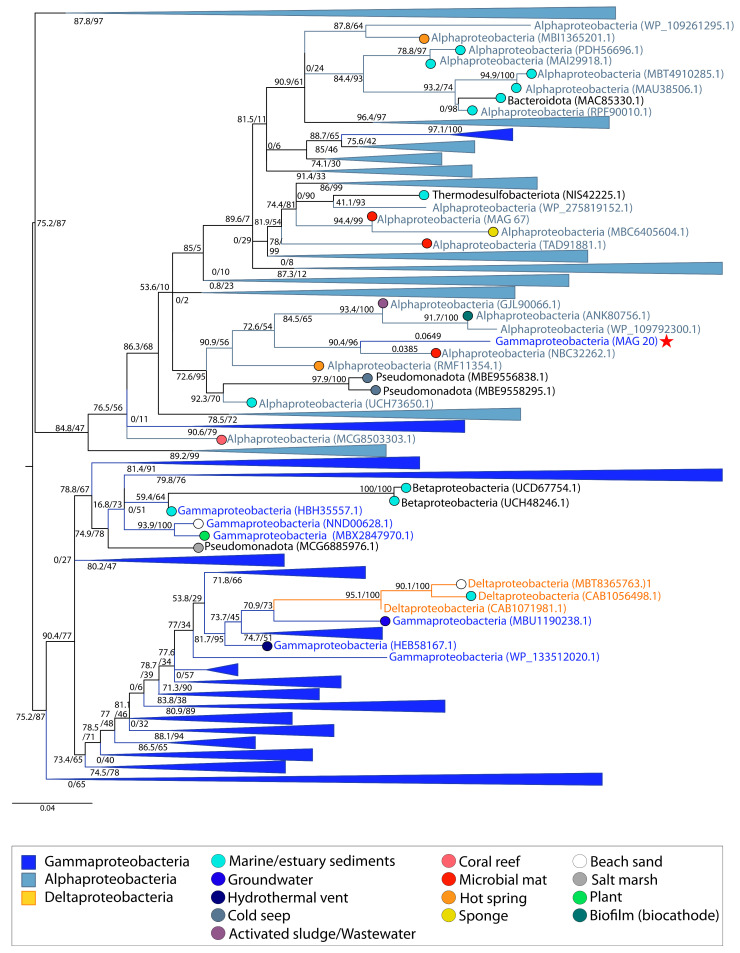
Midpoint-rooted maximum likelihood tree of rubrerythrin (RBR) homologs. Support values indicate approximate likelihood ratio test (aLRT)/bootstrap (100 replicates). The full tree contains 331 unique sequences. Collapsed groups are labeled with taxonomic group names and are colored according to the legend. Legend shows the colors of circles on branch tips according to the sample origin, when data was available. MAGs from the Shark Bay microbial mat metagenome are identified in parentheses as ‘MAG X’. Predicted HGT events involving these MAGs are delineated by a red star. Branch lengths for taxa involved in HGT events are listed above the leaves.

MetaCHIP also inferred HGT of RBR between two Alphaproteobacteria and two Gammaproteobacteria *within* the peritidal microbial mat community (Table S1). The phylogeny of RBR revealed that these transfers did not occur between Proteobacteria within the Shark Bay community (Figure 1D). RBR sequences from Gammaproteobacteria comprised the most basal group with the exception of two more shallow Deltaproteobacteria and Betaproteobacteria clusters. An additional group contained sequences from Alphaproteobacteria. Sequences of Gammaproteobacteria, Bacteroidota, and Thermodesulfobacteriodota nested within this group suggested a history of HGT events between Alphaproteobacteria and these phyla (Figure 7). RBR from Alphaproteobacteria MAG 67 was present within this cluster and thus interpreted as vertically inherited (Figure 1E). RBR from Gammaproteobacteria MAG 20 also resided within this group and was sister to an RBR of an Alphaproteobacterium (NBC32262.1) from a microbial mat community from Bridger Bay in Great Salt Lake, Utah, USA (Figure 7). This indicated the likely transfer of this gene in similar surface-attached communities, yet from various locations. In fact, most of the catalase-peroxidase and RBR gene sequences were identified in organisms from saline systems and biofilm communities (i.e., hypersaline microbial mats, marine sediments, estuary sediments, hydrothermal vents, cold seeps, activated sludge, coral reefs, hot springs, marine sponges, salt marshes, and biocathode biofilms).

**Figure 8:**
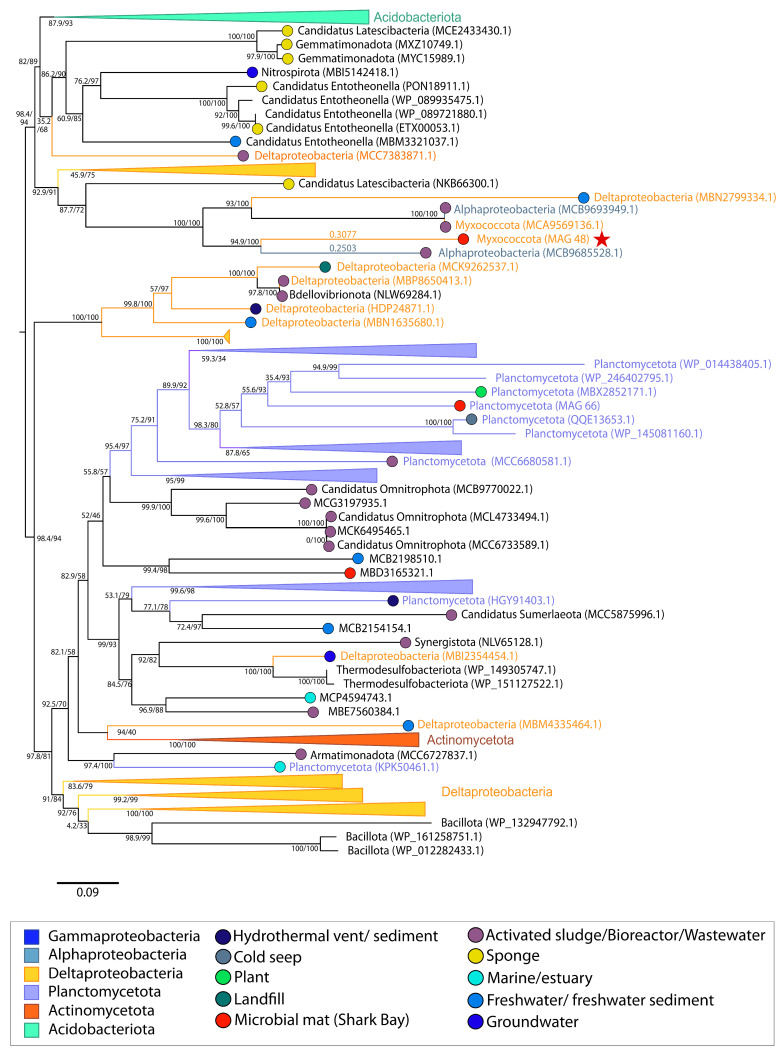
Midpoint-rooted maximum likelihood tree of chaperone protein (*clpB*) homologs. Support values indicate approximate likelihood ratio test (aLRT)/bootstrap (100 replicates). The full tree contains 156 unique sequences. Collapsed groups are labeled with taxonomic group names and are colored according to the legend. Legend shows the colors of circles on branch tips according to the sample origin, when data was available. MAGs from the Shark Bay microbial mat metagenome are identified in parentheses as ‘MAG X’. Predicted HGT events involving these MAGs are delineated by a red star. Branch lengths for taxa involved in HGT events are listed above the leaves.

Molecular chaperone *clpB* encodes a highly conserved heat shock protein with the role of stabilizing proteins and assisting in protein refolding following damage by oxidative stress, heat, low pH, changes in osmolarity, nutrient starvation, etc. [58,59,60,61]. This *clpB* gene was also indicated by MetaCHIP to be transferred between Planctomycetota MAG 66 and Myxococcota MAG 48 (Table S1). Maximum-likelihood phylogeny of *clpB* revealed a polyphyletic sequence distribution that is indicative of multiple HGT acquisitions from different donor lineages. The sequence from Planctomycetota MAG 66 was placed within a Planctomycetota cluster indicating vertical gene transfer (Figure 1E), whereas the sequence from Myxococcota MAG 48 was sister to a sequence from an Alphaproteobacterium (MCB9685528.1) from activated sludge (Figure 1D and Figure 8). The identification of these sequences in MAGs from two different phyla as sister to one another suggests horizontal transfer of *clpB* between these two organisms (Figure 8), although the absence of a clear outgroup prevents us from establishing the direction of HGT. The fact that these proteins that are sister were not both from MAGs from the pustular mat community dataset (Figure 1D) reveals that this HGT event did not occur within the pustular mat community.

### 3.3. Arsenic Toxicity

**Figure 9:**
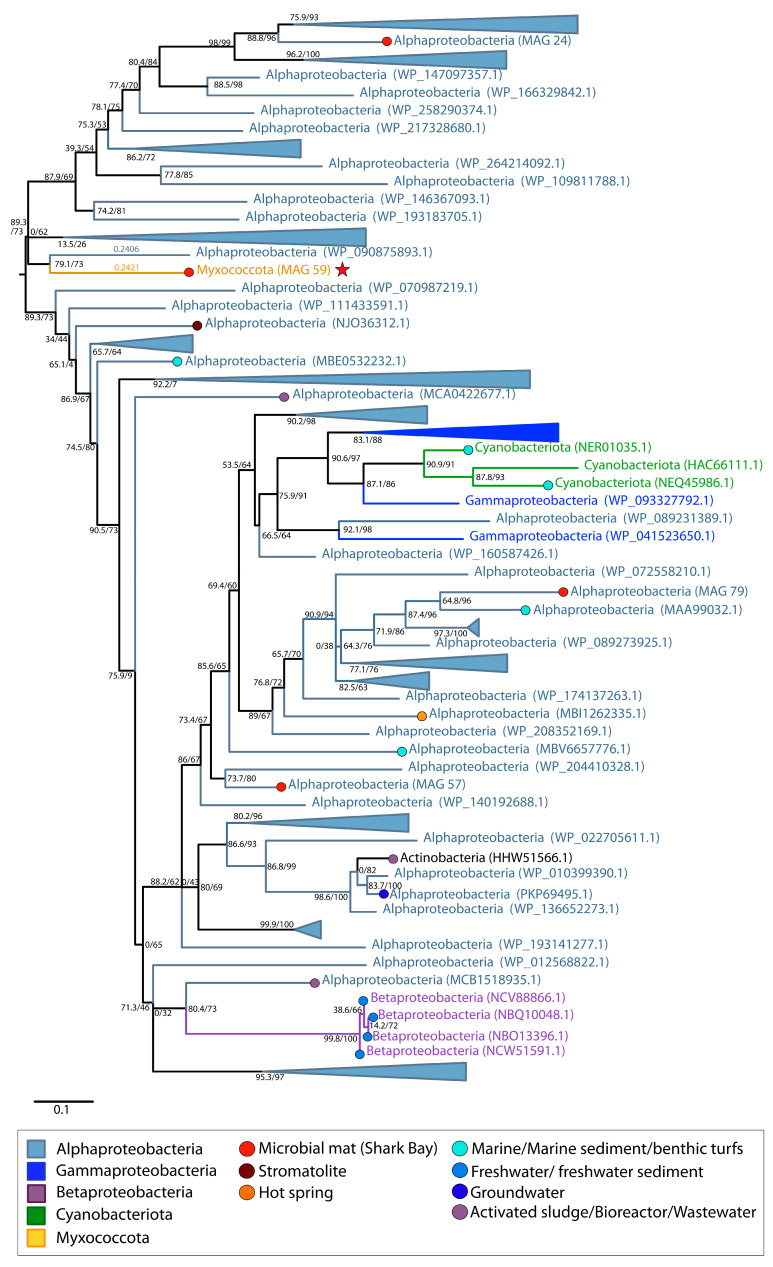
Midpoint-rooted maximum likelihood tree of arsenate reductase (*arsC*) homologs. Support values indicate approximate likelihood ratio test (aLRT)/bootstrap (100 replicates). The full tree contains 212 unique sequences. Collapsed groups are labeled with taxonomic group names and are colored according to the legend. Legend shows the colors of circles on branch tips according to the sample origin, when data was available. MAGs from the Shark Bay microbial mat metagenome are identified in parentheses as ‘MAG X’. Predicted HGT events involving these MAGs are delineated by a red star. Branch lengths for taxa involved in HGT events are listed above the leaves.

Heavy metals, such as arsenic, can displace essential metal ions in metalloenzymes, disrupt protein folding, inhibit enzymatic activity due to similar chemistry to enzyme substrates and oxidize amino acids [62,63,64]. The hypersaline waters in Shark Bay contain some arsenic (5.0 µg/L; [7]), and arsenic-related genes (e.g., *arsC*, *acr3*, etc.) in pustular, smooth, and columnar microbial mat metagenomes from Shark Bay indicate the importance of arsenic detoxification and metabolism in this environment [16,65]. MetaCHIP identified the transfer of *arsC* between Myxococcota MAG 59 and three Alphaproteobacteria (i.e., MAG 24, MAG 57, MAG 79; Table S1). *ArsC* enables the detoxification of arsenate [As(V)] by reduction to arsenite [As(III)]. Maximum-likelihood phylogenies of these proteins recover the monophyly of alphaproteobacterial sequences with the exception of some Cyanobacteria from marine environments, Gammaproteobacteria from unidentified locations, Betaproteobacteria from freshwater localities and one Actinobacteria from an activated sludge source (Figure 9). Homologous sequences of *arsC* from Alphaproteobacteria were identified in multiple environments including stromatolites, hot springs, groundwater, activated sludge from bioreactors, wastewater treatment plants, marine sediments and benthic turfs (Figure 9). The *arsC* of Myxococcota MAG 59 was sister to a sequence identified from an Alphaproteobacterium (WP_090875893.1) and was nested within a group of Alphaproteobacteria (Figure 1D and Figure 9). This suggests the likely direction of transfer of this arsenate reductase from an Alphaproteobacterium to Myxococcota MAG 59 and highlights that this HGT event did not occur between organisms within the pustular mat community.

**Figure 10:**
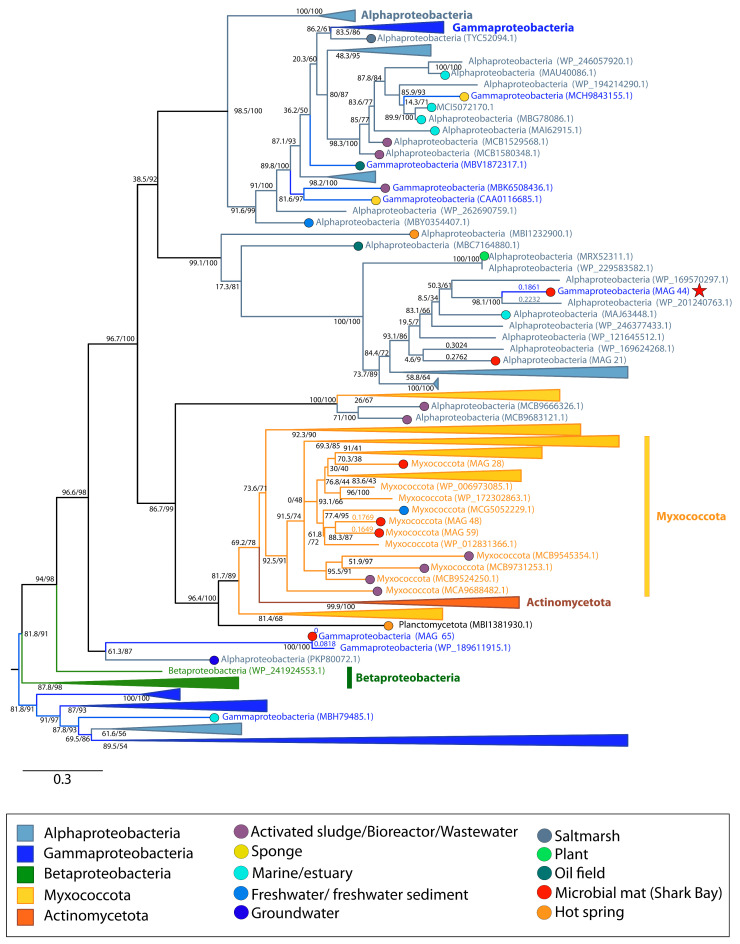
Midpoint-rooted maximum likelihood tree of arsenite efflux permease (*acr3*) homologs. Support values indicate approximate likelihood ratio test (aLRT)/ bootstrap (100 replicates). The full tree contains 413 unique sequences. Collapsed groups are labeled with taxonomic group names and are colored according to the legend. Legend shows the colors of circles on branch tips according to the sample origin, when data was available. MAGs from the Shark Bay microbial mat metagenome are identified in parentheses as ‘MAG X’. Predicted HGT events involving these MAGs are delineated by a red star. Branch lengths for taxa involved in HGT events are listed above the leaves.

The arsenite efflux transporter, *acr3*, is involved in the subsequent step for arsenic detoxification and extrudes As(III) from the cell. MetaCHIP analyses suggest the transfer of *acr3* among Gammaproteobacteria, Myxoccocota and Alphaproteobacteria (Table S1). Maximum-likelihood phylogeny of this gene traces a more complex story. All sequences of *acr3* in Myxococcota MAGs from Shark Bay fall within a cluster of Myxoccocota sequences from various habitats, including freshwater sediment and activated sludge (Figure 10), suggesting the vertical inheritance of this gene within Myxoccocota (Figure 1E). *acr3* of Alphaproteobacteria MAG 21 also groups with other Alphaproteobacteria of unidentified origins. However, *acr3* from Gammaproteobacteria MAG 44 is sister to that of Alphaproteobacterium (WP_201240763.1), potentially suggesting the transfer of this gene from an Alphaproteobacterium to this Gammaproteobacteria MAG (Figure 1D and Figure 10). The placement of Myxoccocota MAG 48 and Myxoccocota MAG 59 as sisters suggest the vertical inheritance of *acr3*, as these MAGs share common taxonomy. 

### 3.4. Relative Timing of HGT Events

**Figure 11:**
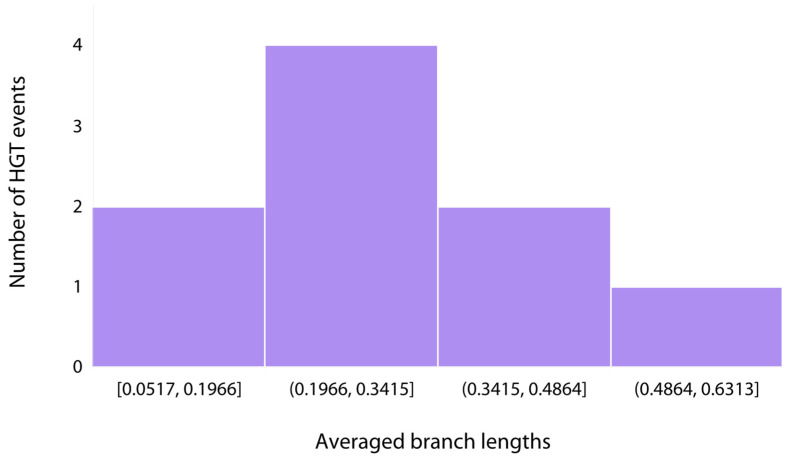
Histogram of branch length distributions for MAGs involved in the HGT of stress-related genes.

One of the challenges to understanding the adaptation and evolution of modern pustular mat communities is being able to distinguish genetic diversity that arose through gene transfer events that predate this system from genes that were acquired through more recent HGT in the mat itself. Molecular clocks are valuable tools for estimating the timing of evolutionary events because they can map the genetic divergence of genes or proteins and estimate divergence times using fossil calibrations that place chronological constraints on certain divergences within a phylogenetic tree [66,67,68]. However, molecular clock models are difficult to apply to single genes for which calibrations are also lacking. In the absence of such models, branch lengths still provide relative age information for specific lineages, including those having undergone HGT. In such cases, the divergence of the reticulating branch places a relative older-bound on the time of transfer, while the diversification of the recipient lineage provides a relative younger-bound constraint (in cases where the reticulating branch is terminal, the younger-bound constraint is essentially *t* = 0). Between these bounds, the exact point of transfer cannot be inferred; therefore, shorter reticulating branches provide more precise dating of HGT events than long reticulating branches. Consistently short (i.e., ~0.05–0.1) lengths of terminal or near-terminal reticulating branches indicate relatively recent HGT events within these trees. While long branches may also contain recent transfer events, these are obscured by a lack of sampling within the HGT donor lineage. Therefore, the distribution of reticulating branch lengths can inform the expected frequency of recent HGT events within gene tree histories. Recent HGT histories can also be inferred for HGT pairs where recipient groups include mat MAG sequences, nested within other mat MAG sequences that constitute the donor lineage. To gain insight on relative timing of these gene transfers, we calculated the branch lengths of each stress-related HGT event and found that the phylogenies of HGT events across all trees revealed a broad distribution of sister branch lengths ranging from 0.0517 to 0.51315 (Figure 11). The average branch lengths between sister branches for *acr3*, *arsC*, GSMT, *opuE*, catalase-peroxidase, trehalase, trehalose transport, *clpB*, and RBR genes were ~0.20, ~0.24, ~0.36, ~0.51, ~0.37, ~0.28, ~0.17, ~0.28, and ~0.05, respectively (Table S2).

## 4. Discussion

Benthic microbial communities, and pustular mats specifically, have adapted to environmental stresses for billions of years [8,9,69,70]. Reconstructing the HGT of stress-related genes between organisms in a modern microbial mat is one mechanism by which we can observe these processes. In light of the expected increase of HGT events associated with biofilm communities [19], the identification of many HGT events in 83 MAGs from a pustular mat in Shark Bay is unsurprising. More frequent exchange of genetic information and faster rates of evolution are also hypothesized in microbial communities from extreme environments relative to the communities that do not experience multiple and consistent stresses [71]. While only 1 of the 89 stress-related genes is consistent with HGT that occurred in the analyzed pustular mat, each of these phylogenies suggest a rich history of ancestral interactions within various environmental settings. MetaCHIP was able to construe gene acquisition events; however, additional phylogenetic analyses clarified some of these HGT predictions as vertical gene transfers and filled in the gaps to reveal a slew of transfer histories that likely occurred prior to the establishment of these modern communities. It is also important to acknowledge that the sequenced metagenome may not represent the entire mat community, and it is possible that genes involved in HGT within the mat are present in unrecovered organisms. Such a scenario would result in the failure to detect some HGTs within the pustular mat.

### 4.1. Adaptations to Osmotic Stress

Microbial adaptations to environmental stresses may be key to the ecological success of specific groups and taxa in these environments. The inferred transfer and acquisition of *opuE* between Planctomycetota and Myxococcota in the pustular mat from Shark Bay illustrates the ongoing adaptations of these organisms to environmental stresses. Phylogeny of *opuE* supports this gene transfer as the only potential HGT event that occurred at the taxonomic class level within the sequenced pustular mat community. The direction of transfer of *opuE* between Planctomycetota MAG 7 and Myxococcota MAG 28 cannot be resolved. However, the identification of this HGT event reveals the potential interaction of Planctomycetota and Myxococcota within Shark Bay. The close relationship in this tree between the *opuE* sequence in Planctomycetota MAG 7 and Myxococcota MAG 28 can most simply be explained by the transfer of this gene within the mat. It is worth noting that a less parsimonious hypothesis can accommodate deeper ancestry of this gene, where Planctomycetota and Myxococcota independently acquired *opuE* earlier in their histories. While less likely, this hypothesis cannot be ruled out. 

The transfer and retention of a trehalose transporter by Gammaproteobacteria (MAG 65) from another marine-associated Proteobacterium demonstrates the importance of this gene within saline environments. Furthermore, trehalase (E.C.3.2.1.28) enzymes catalyze the hydrolysis of the α-glucosidic O-linkage of the sugar trehalose, producing α- and β-D-glucose monomers which can be recovered as a carbon source following decreasing osmolarity [72]. The transfer of trehalase does not enable Gammaproteobacteria (MAG 20) to import and use trehalose as a compatible solute, but it could allow this organism to leverage environmental stress responses of other community members for its own energy production. The acquisition of both a trehalose transporter and trehalase-encoding genes by Gammaproteobacteria MAGs within this pustular mat community suggests the potential exposure of this group of organisms to this metabolite in pustular mats.

The patchy distribution of GSMT indicates a pattern of horizontal inheritance over time, although only a few MAGs predicted by MetaCHIP to transfer GSMT showed clear phylogenetic evidence of HGT events. In such cases, the placement of the GSMT sequences from these MAGs within other phyla (i.e., Myxoccocota MAG 28 within a Planctomycetota cluster) shows that organisms represented by MAGs may have a history of interaction with other organisms outside of this environment. Given that these types of inferences are necessarily limited by the availability of sequences, only deeper sampling and sequencing efforts across many environments may identify the origins of GSMT sequences in the organisms from Shark Bay. Additionally, the phylogeny of GSMT recovers sequences from Verrucomicrobiota (MAG 8) and Candidatus Omnitrophota (MCA9411368.1) as sisters, suggesting interaction between Verrucomicrobiota and Candidatus Omnitrophota.

### 4.2. Adaptations to Oxidative Stress

Photosynthetic microbial mats in peritidal environments undergo UV exposure and oxidative stress [1,73]. The transfer and acquisition of bifunctional catalase-peroxidases is especially beneficial for organisms exposed to oxidative stress, as these antioxidant enzymes can exhibit both catalase (EC 1.11.1.6) and peroxidase (EC 1.11.1.7) activity, dismutating a variety of ROS species including hydrogen peroxides and a broad range of peroxides (ROOH) [54,74]. The acquisition and maintenance of this gene by Chloroflexi MAG 17 suggests the exposure of this Chloroflexi within the microbial mat community to oxidative stress. Notably, neither MAG 17 nor the other Chloroflexi MAG within the mat possesses any other catalase-peroxidase genes, underscoring the potential significance of this acquisition. The identification of RBR within pustular mats from Shark Bay and multiple other hypersaline microbial mats also suggests the potential importance of this antioxidant in mats [75,76,77,78,79]. The identification of the HGT of this RBR mainly throughout environments associated with biofilms (Figure 7) further supports this hypothesis. RBR avoids the production of oxygen by other antioxidant enzymes such as catalase and superoxide dismutase and has thus been implicated in the response of anaerobic bacteria to peroxide stress [80,81,82]. The transfer of this gene throughout multiple biofilm environments where oxygen concentrations likely vary (e.g., anaerobic digester, beach sand, microbial mat, hydrothermal vent, hot spring, sponge, coral reef) suggests that this enzyme may confer benefits on organisms that reside in surface-attached communities that are exposed to changing redox conditions. The presence of this gene in Gammaproteobacteria MAG 20 from Shark Bay may serve to restrict oxygen exposure for this organism, which could potentially reside in an anoxic or micro-oxic niche. The presence of genes involved in both aerobic (i.e., cytochrome c oxidase) and anaerobic (i.e., *adh*) metabolism in this MAG support this inference. The transfer of *clpB* points to additional adaptations of pustular mats to oxidative and other stresses. Phylogeny of *clpB* indicates an extensive history of HGT of this gene in multiple marine and biofilm-associated environments, likely because of its essential nature (Figure 8).

### 4.3. Adaptations to Arsenic Toxicity

Some of the earliest microbial communities are hypothesized to have cycled arsenic [83,84]. Based on the phylogeny of the arsenate-detoxifying *arsC* (Figure 9), the acquisition of *arsC* by Myxococcota MAG 59 from Shark Bay identifies the only reported transfer of this gene to a member of the Myxococcota phylum. Additionally, the history of arsenite efflux permease, *acr3*, appears to be rooted in Gammaproteobacteria, but the gene was later transferred to Myxococcota, Betaproteobacteria, Actinomycetota, Planctomycetota and finally to Gammaproteobacteria MAG 44 (Figure 10). This MAG has only one *acr3* gene (the object of HGT) and *arsC*, so the acquisition of this gene may have conferred the ability of this Gammaproteobacteria MAG to reduce arsenate and extrude the resulting arsenite, increasing its tolerance to arsenic.

### 4.4. Ecological Context of HGT Events

By identifying the ecological context for HGT events involving MAGs sequenced from the pustular mat, we can gain insight on the location and relative timing of these transfers. With the exception of the HGT of *opuE*, between two members of the pustular mat community, all other HGT events occurred outside of the Shark Bay community and did not have outgroups that shared an environmental origin. However, many of these phylogenies did consist of protein sequences from environments that harbor biofilms (e.g., [5,85,86,87,88,89,90,91]). This may suggest that the transfer of these particular stress-response genes is particularly beneficial within biofilm communities. Because a majority of these transfer events did not occur within the pustular mat from Shark Bay, it is likely that these HGTs occurred prior to the formation of this mat, but in some surface-attached community.

### 4.5. Relative Timing of Gene Transfers and Identification of Relationships among Microbes in Complex Communities

The broad distribution of branch lengths involving HGT events (Figure 11) suggest that some transfers occurred more recently, leading to shorter branches and others either occurred deeper in time or there is poor sampling depth as a result of a lack of sequence availability, so the branches in phylogenetic trees leading to them are longer. While the older bounds on these HGTs cannot be constrained, shorter branch lengths can provide more precise timing of these events. The potential transfer of the RBR gene between Gammaproteobacteria MAG 20 and an Alphaproteobacterium (NBC32262.1) involved the shortest averaged branch lengths which suggests that this transfer likely constituted the most recent HGT event among the stress-related genes observed. The transfer of the trehalose transporter between Gammaproteobacteria MAG 65 and an Alphaproteobacterium (MBC6405981.1) constituted the second most recent HGT transfer; however, the disparities in localities underscore that this event likely occurred prior to the establishment of Gammaproteobacteria MAG 65 in the microbial mat (Figure 5B). In fact, the majority of phylogenies depicting potential HGT events at the class level indicate that these events likely occurred before the organisms from Shark Bay were established within these pustular mats. This suggests that several of the organisms within the microbial mat were preadapted to the stressors they face in Shark Bay, although further analyses at the species level, for instance, can offer additional insights.

The identification of HGT events among organisms can also offer additional insights into microbial interactions which can be otherwise difficult to decipher within complex communities. These gene transfer events can serve as a snapshot of a past interactions among microbes. The specific identification of the transfer of *opuE* between the Planctomycetota and Myxococcota MAGs in Shark Bay reveals the potential interaction of these two organisms within this pustular mat. All other HGT events recorded between organisms inside and outside of the pustular mat community may also point to previous interactions of these taxonomic classes in localities that are currently difficult to constrain. We would have a higher degree of certainty about the locations of these transfers and participating organisms in these HGT events only if every existing and pre-existing organism were sequenced, and sequenced perfectly at that. Despite limitations in available sequence data, these HGT detection and phylogenetic analyses methods provide valuable tools for uncovering microbial interactions across diverse environments. They are particularly potent when applied within biofilm communities, where organisms are sessile and HGT is abundant.

### 4.6. Augmenting ‘Stand-Alone’ HGT Detection Methods

Overall, MetaCHIP and similar HGT-detection programs are important tools for detecting HGTs within uncultivable microbial communities for which only MAGs exist. These approaches can recognize genes that were likely horizontally transferred between members of different phyla, classes, orders, etc. This study indicates high rates of false positives which are likely not specific to stress-related genes. For this reason, hypotheses developed using MetaCHIP and similar HGT-detection programs should be augmented by phylogenomic relationships that use as many publicly available sequences as possible to ‘fill’ the tree space. Supplementing these HGT prediction tools with additional phylogenetic analyses will provide a more accurate, fine-scale resolution of these potential HGTs and can even provide insight into the relative timing and location of these transfers.

## Data Availability

MAGs used in these phylogenetic analyses are publicly available on Zenodo (https://doi.org/10.5281/zenodo.3874996, accessed 28 November 2023) and on the Joint Genome Institute website under the GOLD AP ID Ga0316160.

## Supplementary Materials

The following supporting information can be downloaded at: https://www.mdpi.com/article/10.3390/genes14122168/s1, Figure S1: Relative HGT events predicted between relative number of MAGs belonging to each phyla; Figure S2: Categorized genes involved in HGT events as predicted by MetaCHIP; Table S1: Predicted HGT genes related to stress response; Table S2: Average branch lengths of horizontally transferred genes.
