# Supplementary figures and images for: Assessing the Influence of HGT on the Evolution of Stress Responses in Microbial Communities from Shark Bay, Western Australia
Source: Genes (Basel). 2023 Dec 1;14(12):2168. doi: 10.3390/genes14122168 (PMC10742547; doi:10.3390/genes14122168)

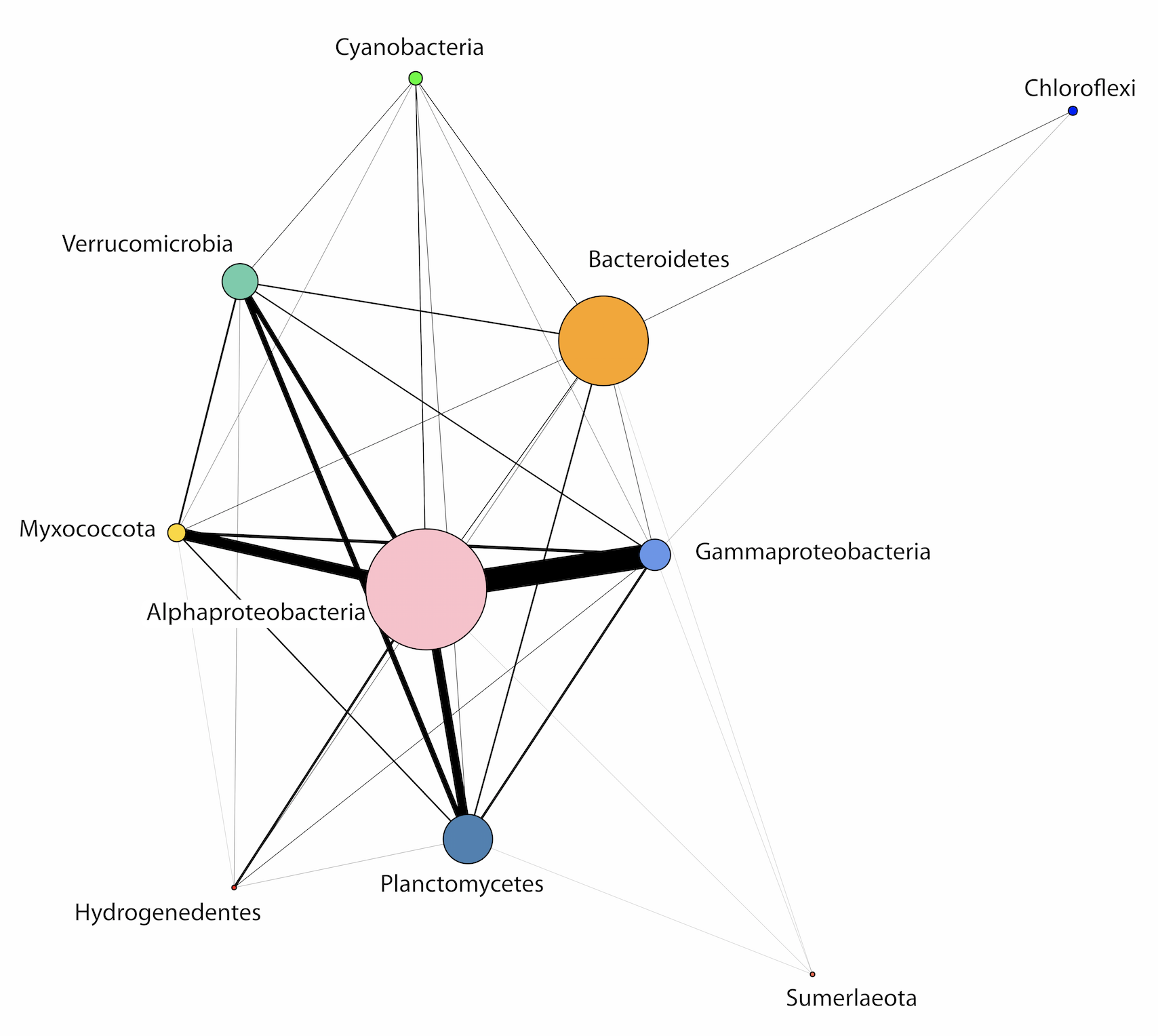

Supplement: Supplementary file 1 [file genes-14-02168-s001.zip › Figure_S1.png]

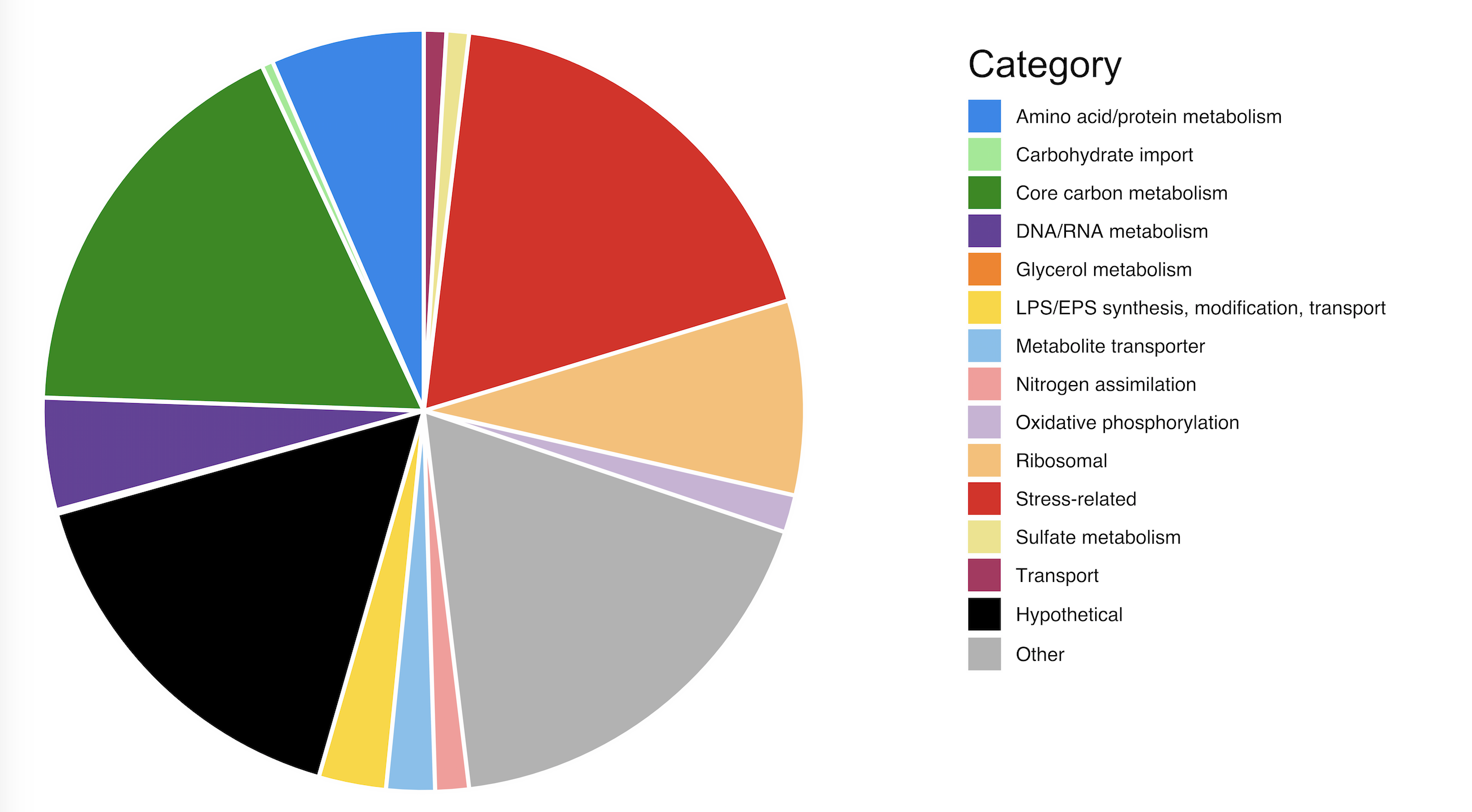

Supplement: Supplementary file 1 [file genes-14-02168-s001.zip › Figure_S2.png]
